# Give Peace a Chance? How Regulatory Foci Influence Organizational Conflict Events in Intractable Conflict Environments

**DOI:** 10.1177/01492063231196556

**Published:** 2023-09-07

**Authors:** Libby Weber, Angelique Slade Shantz, Geoffrey M. Kistruck, Robert B. Lount

**Affiliations:** University of California, Irvine; 3158 University of Alberta; York University; 2647 The Ohio State University

**Keywords:** organizational conflict, intractable conflict, intractable conflict environment, regulatory focus, field experiment

## Abstract

An intractable conflict environment (ICE) is an extreme context in which deep, unsolvable conflict between groups is central to the actors within it. While non-ICEs are typically assumed in organizational research, ICEs are increasingly common contexts for organizations. Moreover, this context influences peoples’ interpretation of potential organizational conflict incidents inside the organization and therefore the likelihood and emotional intensity of organizational conflict events. Whereas a potential conflict incident, such as a disagreement over how to complete a task, may be perceived as benign in a more typical environment, the same incident is more likely to be interpreted as much more negative and emotionally intense when taking place in an ICE, increasing the frequency of conflict events (conflictual behavior). Prior work suggests that, in a typical environment, promotion-framed (achieving positives) interventions reduce conflict more than prevention-framed (avoiding negatives) interventions by temporarily inducing promotion orientations that reduce the likelihood of interpreting conflict. However, we argue an ICE induces a strong prevention focus, which overrides promotion-framed interventions. Instead, we argue in an ICE, a prevention- rather than promotion-framed intervention is likely to be more effective because it “matches” the strong prevention focus. To test this prediction, we examine the difference in number of conflict events in farming cooperatives in rural Ghana (an ICE) after instituting prevention- versus promotion-framed interventions aimed at addressing conflict. Quantitative and qualitative findings from a 9-month field experiment support our hypothesis.

“Prevention is better than cure.”

—Benjamin Franklin, and interviewee, Sabare Village

## Introduction

An ICE is an extreme context in which deep, unsolvable conflict between groups, whose members have rigid, polarized collective identities constructed around opposing views (Coleman, 2003), is central to the actors within it. The enduring conflict in this context is seen as a zero-sum game (Bar-Tal, 2007), with one group's beliefs negating those in the other. As such, these unsolvable conflicts typically stem from antithetical differences, such as land disputes (e.g., the century-old Palestinian-Israeli conflict), political views (the extreme political environment in the United States arising from polarization of Democrats and Republicans), or religious beliefs (the highly emotional and often violent conflict that occurred between Protestants and Catholics in Ireland). Each group's beliefs or views pose a direct threat to the other group's collective identity, which results in high-intensity negative emotions such as fear and anger, and negatively impacts the perceptions, emotions, and behaviors of the individuals within the extreme context (e.g., Shmueli, Elliott, & Kaufman, 2006).

The ICE's strong influence on perceptions, emotions, and behaviors is particularly significant because Pondy's (1967) model of organizational conflict is based on these three elements. His model starts with a *potential conflict incident*, an act by one party that arises from task, relationship (De Dreu & Weingart, 2003), or role differences (Pondy, 1967) and hinders another's resources, autonomy, or goals. For example, if project groups A and B each need 100,000 semiconductors, but only 100,000 are delivered and taken by project manager B, group A's production is hindered by the other party. This incident is then either perceived by group A members as benign, leading to neutral emotions, or conflictual, invoking negative emotions and a *conflict event*, “any of several varieties of conflictual behavior” (Pondy, 1967: 303).

Given the ICE's strong influence on perceptions, a potential conflict incident is likely perceived differently in an ICE versus a non-ICE (Felstiner, Abel, & Sarat, 1980). Specifically, an ICE threatens the organizational actors’ collective identity related to the intractable conflict, invoking high-intensity negative emotions and inducing a strong focus on avoiding any negative events (a prevention focus in regulatory focus theory [Higgins, 1997, 1998]). Because the cues from the ICE are more salient than less valanced and less comparably intense situational cues or general dispositional tendencies, this strong prevention focus negatively colors perception of potential conflict incidents, casting them as intentional and harmful. In the example, group A members in an ICE would tend to perceive project manager B's actions as intentional, increasing the chance of conflict events. In contrast, because a non-ICE does not threaten organizational actors’ identities, the more neutral organizational environment tends to shape actors’ perceptions of the potential conflict incident as benign. Here, group A members are less likely to view manager B's actions as intentional, invoking more neutral emotions that are less likely to lead to a conflict event. So, even though an ICE is *outside* of organizations, it increases the likelihood that a potential conflict incident is perceived as conflictual (and that a conflict event actually arises) *inside* organizational boundaries, while a non-ICE does not. As a result, compared to a non-ICE, conflict events in an ICE (1) are more frequent and (2) are prompted by more intense negative emotions (e.g., Fiol, Pratt & O’Connor, 2009; Pondy, 1992).

Work on conflict interventions in the organizational behavior (Jehn, 1995; Pondy, 1967) and cognitive governance (Williamson, 1985) literatures typically assumes a non-ICE, suggesting these traditional interventions may not adequately address conflict events in an ICE. Given that conflict events are much more likely, and the resulting emotions more intense and negative in an ICE than a non-ICE, these conflictual behaviors are more harmful to organizations and should be addressed ex ante rather than ex post. Yet, the organizational conflict literature typically focuses on ex post management of conflict events (e.g., Gilin Oore, Leiter, & LeBlanc, 2015), which is likely inappropriate in an ICE. In contrast, cognitive governance research draws on regulatory focus theory (RFT) (Higgins, 1998) to address organizational conflict ex ante, using contract frames to invoke a *situational* regulatory focus (Weber & Mayer, 2011) that shapes perceptions, emotions, and behaviors. Specifically, these studies predict a promotion-framed contract induces a promotion focus (focus on achieving positives), reducing the likelihood of perceiving incidents as conflictual (Crowe & Higgins, 1997; Weber & Bauman, 2019) as compared to a prevention-framed contract.

Yet, when an organization is in an ICE, we argue a promotion-framed intervention is less likely to be effective than in a non-ICE. An ICE threatens the organizational actors’ collective identity and (often) safety, strongly focusing their attention on preventing any negative consequence, even those unrelated to the enduring conflict. This prevention focus—stemming not from a chronic and immutable dispositional regulatory focus nor from the momentary potential conflict incident, but rather from the enduring extreme environment—is very strong and highly salient to the actors in an ICE, so it is unlikely to allow the induction of a situational promotion focus by the framed intervention. As a result, the effectiveness of a promotion-framed intervention is limited in an ICE.

Given that neither traditional organizational conflict nor cognitive governance interventions are likely to be adequate in an ICE, here we ask: How can organizations address conflict events in an ICE ex ante? An effective intervention in an ICE must take into account the strong prevention focus induced by the extreme environment and the increased likelihood that individuals will perceive incidents as conflictual. Regulatory fit theory (Cesario, Higgins, & Scholer, 2008; Higgins, 2000), a companion to RFT, focuses on the feeling of “rightness” that occurs when an actor's regulatory focus matches their means to pursue a goal (Lount, Pettit, & Doyle, 2017). This approach suggests a different avenue to address conflict events ex ante, lowering the likelihood of conflictual behavior by getting people to do what feels “right.” Thus, we predict that matching the strong prevention focus (induced by the ICE) with a prevention-framed intervention decreases conflictual behavior, reducing the incidence of conflict events. That is, the prediction in an ICE is opposite that in a non-ICE.

To test this hypothesis, we undertook a 9-month field experimental study within cooperative organizations in Northern Ghana, which has been plagued by periods of intractable conflict for decades, including the timeframe of our study. We designed two versions of an intervention, one prevention-framed (avoiding negative behavior) and one promotion-framed (promoting positive behavior). We randomly assigned cooperatives to one of these two interventions and measured the number of conflict events involving cooperative members in the following months. Our hypothesis, that the prevention-framed intervention is associated with fewer conflict events than the promotion-framed intervention, was supported, corroborating our underlying assumption that the extreme environment of intractable conflict induces a prevention focus, and that this leads to more effective results from a prevention-framed intervention.

Our study makes three important contributions. First, we identify that in an ICE, the most effective intervention to address conflict events ex ante is the opposite of that prescribed in the non-ICEs typically studied in organizational behavior. Moreover, to address conflict in an ICE, a prevention-framed intervention acts to reduce conflictual behavior, which is distinct from a promotion-framed intervention decreasing the perception of incidents as conflictual in a non-ICE. Relatedly, we expand understanding of the impact of an extreme external environment of intractable conflict—an ICE—on the organization. An ICE tends to increase the likelihood that potential conflict incidents are perceived as conflictual and lead to more intense negative emotions than in a non-ICE. Second, we contribute to regulatory focus and fit theories by proposing a renewed emphasis on the broader environment within which dispositional (individual) and situational (momentary cues) regulatory foci occur. In so doing, we advance understanding of when a regulatory focus may and may not be situationally induced. Finally, we provide practical guidance by suggesting managers use prevention-framed interventions to address conflict events in an ICE. Most attempts to do so are promotion-focused, following John Lennon's advice in his famous song “Give Peace a Chance.” Although counterintuitive, our findings suggest the messaging of “give conflict avoidance a chance” tends to resonate with individuals in an ICE, more successfully addressing conflict events.

## Organizational Conflict Interventions Inside a Non-Intractable Conflict Environment

Although ICEs occur in both developing and developed economies (e.g., Waytz, Young & Ginges, 2014), and thus are often backdrops for organizations, *non-*ICEs are typically assumed in organizational research. In stark contrast to an ICE, a non-ICE is a benign context of nonconflict or mild, short-term solvable disputes, peripheral to the organizational actors’ identities. See Table 1a for a contrast of an ICE versus a non-ICE. It does not invoke high-intensity emotions or directly threaten the actors’ identities, making it unlikely to shape their perceptions, emotions, and behaviors. An archetype of a non-ICE is the national context of Nordic countries, which typically features a balance of power between government and citizens, as well as widespread concern for social justice and providing basic human needs. In these non-ICEs, the organization tends to be a stronger source of influence than the broader environment (Aldrich & Pfeffer, 1976). Table 1b compares organizational conflict events in an ICE versus a non-ICE.

**Table 1a:** Characteristics^a^ of an ICE^b^ Versus Non-ICE

	Environment Characteristics	
	Non-ICE	ICE
*Psychological factors:*		
Ideologies	Nonconflictual views or conflictual views with integrative or distributive solutions	Dominant opposing views; one negates the other
Identities	Adaptable individual identities unrelated to conflictual views	Frozen, polarized collective identities constructed around opposing views
Emotions	Positive to low-intensity negative emotions	High-intensity negative emotions
*Resulting conflict:*		
Complexity	Zero to few isolated points of disagreement	Many interrelated points of opposition
Duration	Short-term disagreement	Enduring animosity
*Impact on future cognition:*		
Strength	Low	High
Attentional focus	None	Avoiding negatives from outgroup and nonconforming ingroup members

Adapted from Coleman 2003 (Table 1, pp. 9–10).

ICE = Intractable Conflict Environment.

**Table 1b:** Perceived Organizational Conflict Characteristics Under an ICE Versus Non-ICE

	Perceived Organizational Conflict Characteristics	
	Non-ICE	ICE
Threat to identity	Low threat to individual identity	High threat to collective identity
Emotions from perceived conflict	Low-intensity negative emotions	High-intensity negative emotions

ICE = Intractable Conflict Environment.

By implicitly presupposing non-ICEs, prior organizational conflict intervention research assumes this benign environment has little impact on the perception of potential conflict incidents. Rather, traditional organizational behavior interventions take conflict events arising from either task or relational issues as given. Task conflict generally enhances organizational performance (Jehn & Mannix, 2001), so this work suggests allowing it to continue while prescribing ex post interventions to mitigate the slightly more negative emotions and conflictual behavior arising from interpersonal issues (e.g., Rahim, 2002).

**Figure 1:**
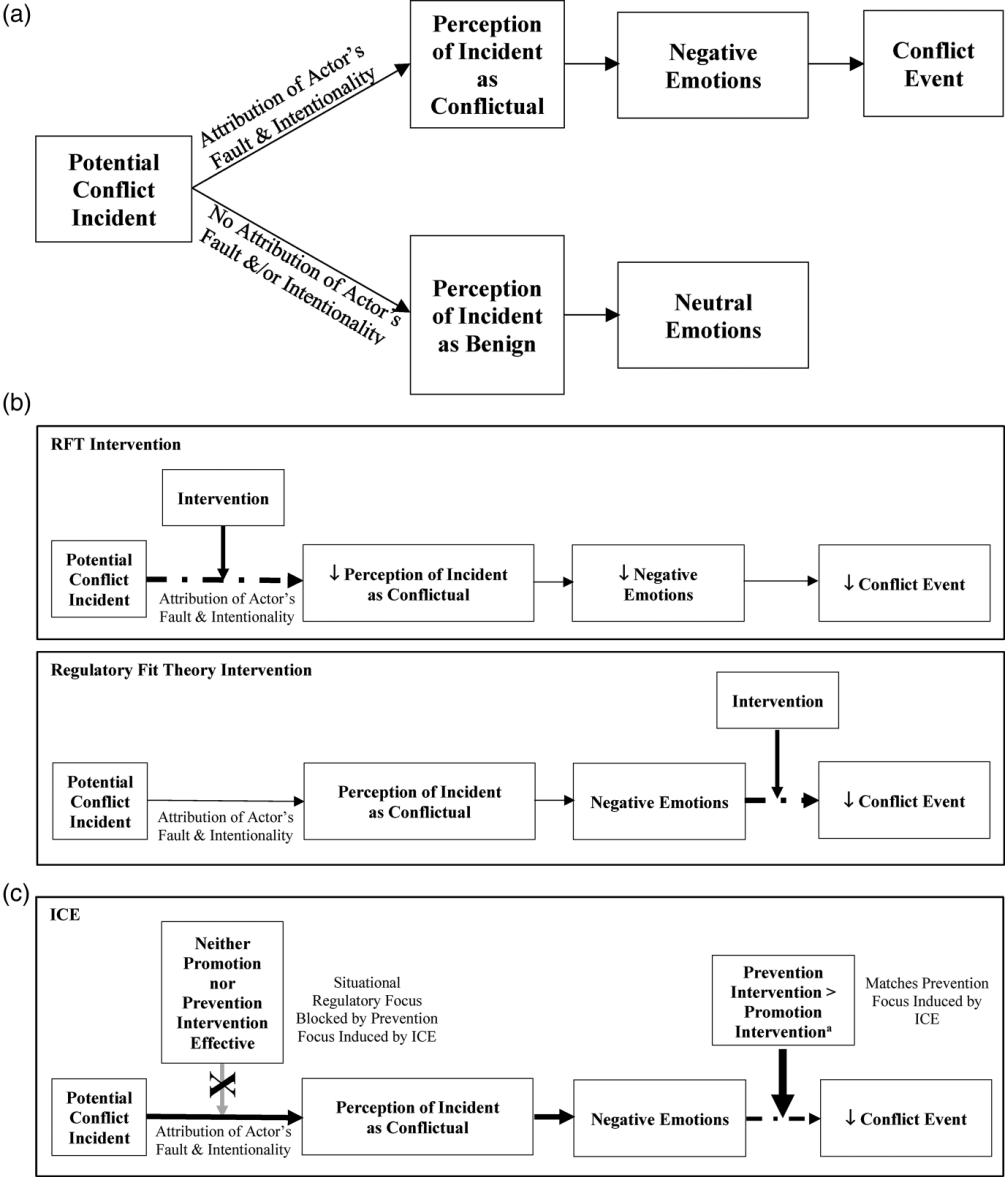
(a) Adaptation of Pondy's (1967) model of organizational conflict. (b) Comparative model of point of action for RFT and regulatory fit theory interventions to address organizational conflict. (c) Comparative model of prevention and promotions interventions in an ICE.

^a^Only point of impact prevention or promotion-framed intervention can have in an ICE because it blocks the intervention's impact on perception of a potential incident as conflict.

In contrast, cognitive governance studies attempt to reduce the perception of potential conflict incidents as conflictual through framing interventions (Weber & Mayer, 2011) based on RFT (Higgins, 1997, 1998). This approach relies on Pondy's model of a conflict event. First, a potential conflict incident occurs, in which the interests of one party are subjugated by another. However, this incident does not become a conflict event unless the disadvantaged party first perceives their interests were intentionally hindered by the other party (Thomas & Pondy, 1977), and the negative emotions that result from this perception prompt conflictual behavior (the conflict event). The incident is more likely to be perceived as conflictual when the act leading to the disadvantaged state is both attributed to the other party and seen as intentional (Louis, 1977; Thomas & Pondy, 1977). Thus, the same potential conflict incident may be interpreted as either conflictual or benign (Felstiner et al., 1980). If it is viewed as conflictual, a conflict event (negative behavior) tends to occur. See Figure 1a for a summary of the conflict event model.

Given the large number of other negative organizational behaviors and events that can occur, it is important to be clear about how a conflict event differs from them. Conflict events are distinct from wrongdoing—a behavior that crosses the line of right and wrong (Schnatterly, Gangloff & Tuschke, 2018: 2408)—as it must be induced by the perception that one party intentionally hindered another's resources, autonomy, or goals. Conflict events are also markedly different from mistreatment—an unfair behavior toward another party (e.g., Boroff & Lewin, 1997)—as mistreatment focuses on a broad class of behaviors that may be perceived as unjust, similar to the potential conflict event. Finally, conflict events are distinct from crisis events—(unexpected situations that lead to mass disruption (Boin, 2004: 167)—as crisis events are negative situations rather than negative behaviors.

### Using Regulatory Focus Theory to Shape Interpretations of Conflict

The cognitive governance approach uses contract frames to influence the perception of a potential conflict incident (e.g., Weber & Mayer, 2011), shaping the attribution and perceived intentionality of the other party's action (Weber, 2017; Weber & Bauman, 2019). This research draws on RFT (Higgins, 1997, 1998), which suggests an individual interprets situations in systematically different ways when viewing them with a prevention versus promotion focus. While individuals may have a general *dispositional regulatory focus* (low or high in prevention and/or promotion; Higgins, 1998), these orientations can also be situationally induced through loss and gain framing (Pham & Higgins, 2005; Roney, Higgins, & Shah, 1995). In other words, framing can be used to induce a momentary *situational regulatory focus*. Under a prevention (loss-framed) contract, individuals view a goal as something that *must* be reached (Brendl & Higgins, 1996). If the goal is missed, they are likely to experience high-intensity agitation. However, if the goal is reached, individuals tend to experience low-intensity contentment. The desire to avoid high-intensity agitation is stronger than the desire to experience low-intensity contentment, invoking vigilance (here “vigilance” refers to a state of high alert toward avoiding potential threats) against negative outcomes (Higgins, 1998). This focus on avoiding negatives also colors perceptions of the other party's neutral behavior such that it is often viewed as self-interested and even opportunistic (Weber & Mayer, 2011).

In contrast, under a promotion (gain-framed) contract, the *same* goal is viewed as an ideal (Higgins, 1997, 1998). If the goal is missed, the party is disappointed (Brendl & Higgins, 1996), which is comparably less negative than the agitation experienced under a prevention focus. But if the goal is achieved, the individual experiences high-intensity happiness, which is more positive than the contentment felt under a prevention contract. The desire to achieve high-intensity happiness is stronger than the desire to avoid low-intensity disappointment, invoking eager behavior to achieve positive events (Higgins, 1998). Also, this focus on achieving positives suggests neutral behaviors are perceived positively, leading other individuals to be viewed as generally cooperative under a promotion contract (Weber & Mayer, 2011).

So, individuals under a prevention-framed or promotion-framed contract are not equally likely to perceive the same potential conflict incident as conflictual. Under a prevention-framed contract, the incident tends to be perceived as a threat, inducing high-intensity agitation and a view of the other party as opportunistic (Weber & Bauman, 2019). Both aspects increase the likelihood the incident is perceived as conflictual (Weber, 2017). In contrast, under a promotion-framed contract, individuals are more likely to perceive the same incident as unintentional (Weber & Mayer, 2011) or attribute it to the situation (Weber & Bauman, 2019). Moreover, if the incident is perceived as conflictual, the disadvantaged individual experiences low-intensity disappointment rather than high-intensity agitation. Thus, the emotions from perceived conflict are less intense and negative under a promotion contract than under a prevention contract. Thus, *in a non-ICE, a promotion contract is less likely to lead to the perception of potential conflict incidents as conflictual than a prevention contract* (Weber & Bauman, 2019).

This literature, set in the context of typical interorganizational and intraorganizational exchanges, does not consider the impact of ICEs. Yet, the context, particularly an extreme environment (Hӓllgren, Rouleau, & De Rond, 2018), has been shown to significantly influence perceptions (Ross & Nisbett, 1991). As a result, the general prescriptions from cognitive governance may not hold in an ICE, suggesting it is necessary to examine how it may impact the likelihood and intensity of conflict events and the success of promotion-framed interventions.

## Organizational Conflict Interventions Inside an Intractable Conflict Environment

Shmueli and coauthors (2006) suggest an ICE acts as a strong cognitive frame, a mental map through which events are interpreted (Weick and Bougon, 1986), even inside the organization. Recent work focusing on the impact of extreme negative outcomes, prevalent in an ICE, addresses this effect. Specifically, extreme negative outcomes, such as harm to individuals’ identity, self-esteem, and safety, are more strongly encoded into their memories than typical events and outcomes. This occurs through “emotional tagging” (Laudenbach, Malmendier, & Niessen-Ruenzi 2019), which makes these extremely negative memories more easily accessible (Malmendier & Wachter, 2021) when the individual is in the environment. Additionally, neuroscience research suggests “that under conditions where the deployment of attentional resources is limited . . . emotional information is prioritized and receives privileged access to attention” (Vuilleumier 2005: 586). Thus, the cues from the ICE are likely to be more salient than less valanced and less comparably intense situational cues or general dispositional tendencies.

When highly salient cues from an ICE trigger these significantly negative memories of loss and threats, they are likely to focus the individual on avoiding the harmful consequences that previously occurred (e.g., Malmendier & Nagel, 2011; Malmendier & Steiny, 2018). As a result, individuals in an ICE tend to be strongly focused on preventing negative outcomes, inducing a strong prevention focus (Higgins, 1998). This prevention focus increases the likelihood that even relatively minor potential conflict incidents, unrelated to the ICE, are perceived as conflictual.

Although RFT suggests a gain-framed intervention induces a promotion focus, this research not only assumed a non-ICE but was primarily conducted in developed, westernized (often laboratory-based) settings (e.g., Crowe & Higgins, 1997; Roney et al., 1995) typified by a benign environment. So, this work does not consider how an extreme environment may limit the situational induction of a promotion focus from a gain-framed intervention.

As we argued previously, the high salience of significant threats (e.g., to self-esteem, identity or even safety) in an ICE tends to induce a prevention focus. Moreover, this tendency to view the world in terms of preventing negatives is likely to be very strong, as it stems from the high desire to avoid extremely negative outcomes in the ICE. The gain-framed stimuli in a promotion intervention are much weaker in comparison. Thus, the strong prevention focus induced by an ICE tends to prevent the situational induction of a promotion focus from a gain-framed intervention, as the individual's attention is drawn to the highly salient threats in the ICE rather than the framing. Thus, in an ICE, a gain-framed intervention is unlikely to shape perception of a potential conflict incident by situationally inducing a promotion focus and therefore tends to be less effective in addressing conflict events than in a non-ICE. Instead, an effective intervention in an ICE must accept this strong prevention focus, the increased tendency to perceive incidents as conflictual, and the resulting high-intensity negative emotions.

### Using Regulatory Fit Theory to Make Vigilant Behavior Feel “Right”

While an intervention based on RFT attempts to change the organizational actors’ perceptions of potential conflict incidents, one based on regulatory fit theory (Higgins, 2000) attempts to change their behavior, reducing the incidence of conflict events themselves. Thus, a regulatory fit intervention does not act to lower the likelihood of perceiving incidents as conflictual but instead reduces the tendency for negative behavior after a conflict is perceived. See Figure 1b for the different points of action of an RFT and a regulatory fit theory intervention.

Regulatory fit theory is an extension of RFT that relies on the same prevention and promotion foci as the original theory. However, regulatory fit theory focuses on how certain behavior feels “right” for reaching a goal. Specifically, the theory argues that when there is a match between the intervention framing and the existing regulatory foci, individuals are more likely to achieve the goal of the intervention (Lount et al., 2017). This occurs because “when people pursue a goal in a manner that sustains (fit) rather than disrupts (nonfit) their current regulatory orientation, people feel right about their goal pursuit activity, and their engagement in the activity is strengthened” (Higgins, Cesario, Hagiwara, Spiegel, & Pittman, 2010: 560). The increased strength of engagement results in greater enthusiasm to perform the behavior and accomplish the goal (Cesario et al., 2008). This is why matching an intervention's frame to a person's regulatory focus makes the message more persuasive to them than if the frame is mismatched (Aaker & Lee, 2006).

Furthermore, regulatory fit increases their feelings of “rightness” about their positive and negative reactions to events. This affirmation is then often transferred to later decision-making, as people imagine the pleasure or pain of the outcomes that particular choices will produce: “Imagining making a desirable choice has higher fit for people in a promotion focus than it does for those in a prevention focus (because success maintains eagerness but reduces vigilance); the opposite is true for imagining making an undesirable choice (because failure maintains vigilance but reduces eagerness)” (Higgins, 2005: 211).

A prevention-framed intervention induces vigilant behavior that matches the need to avoid negatives induced by the ICE. Thus, vigilant behavior to avoid further negative events feels “right” in this environment, lowering the incidence of conflictual behavior (the conflict events themselves) that typically follows the perception of conflict and the resulting negative emotions. That is, the prevention-framed intervention breaks the link between the negative emotions felt after conflict is perceived by inducing vigilant behavior that feels right to avoid a conflict event. Thus, despite an ICE typically increasing the perception of conflict, a prevention-framed intervention is still likely to result in fewer actual conflict events in this extreme environment, as it reduces the negative behavior typically invoked by the perception of conflict.

Conversely, a promotion intervention induces eager behavior (cooperation) to increase positive events (Higgins, 1998), crowding out conflictual behaviors. However, this approach is not as successful at lowering potential conflict events in an ICE, as there is a mismatch between the eager behavior invoked by the intervention and the ICE-induced prevention focus. As a result, the eager behavior does not feel right to individuals in an ICE, even if it is intended to reduce conflict events. This is particularly true because eager behavior is seen as reducing vigilance, which is paramount in this extreme environment. Thus, in an ICE, the individuals are not as enthusiastic about pursuing positives behaviors as they are about avoiding negative behaviors, making a promotion-framed intervention less effective than a prevention-framed intervention at reducing conflict events. See Figure 1c for a summary of the differences between a prevention and promotion intervention in an ICE.

As such, an intervention framed to prevent negative behaviors creates a “fit” with the prevention focus induced by the extreme environment, as compared to an intervention that is framed to promote positive behaviors. Formally:

*Hypothesis 1:* In an intractable conflict environment (ICE), a prevention-framed intervention is likely to be associated with fewer conflict events than a promotion-framed intervention.

## Methodology

### Study Context

Cooperative-based forms of economic organizing (e.g., farming cooperatives, credit cooperatives, worker cooperatives, etc.) are increasingly used in extreme contexts of intractable conflict to increase equity and participation in poverty alleviation efforts (Jones Christensen, Siemsen, & Balasubramanian, 2015; Kistruck, Sutter, Lount, & Smith, 2013; Stephan, Patterson, Kelly, & Mair, 2016). Yet, despite the promise of these cooperative organizational approaches, these organizations often struggle to achieve their goals, due in part to the negative conflict events that are frequently experienced by organizational members (Ashforth & Reingen, 2014; Ben-Ner & Ellman, 2013; Slade Shantz, Kistruck, Pacheco, & Webb, 2020). Thus, this context is particularly well-suited to examine the comparative effectiveness of prevention and promotion-framed interventions at addressing conflict events in an ICE.

We conducted a field experiment in collaboration with Co-op Ghana (a pseudonym), the Ghanaian subsidiary of a regional West African development network operating in Ghana, Liberia, and Sierra Leone. Each subsidiary operates as a national NGO with local management systems to ensure locally tailored initiatives. Co-op Ghana's primary project at the time of our work with them was to reduce poverty by organizing independent farmers into newly formed cooperative groups. Specifically, Co-op Ghana encouraged members of these nascent organizations to share key resources (i.e., land, tractors, grain silo, etc.), coordinate planting and harvesting, store their yields until prices peak for their crops, and pool financial resources through the creation of small cooperative banks.

Our initial engagement with Co-op Ghana began with a series of discussions with leaders and field staff regarding their experiences in establishing local cooperatives within villages in Northern Ghana. During these meetings, Co-op Ghana expressed concerns with the feasibility of continuing to establish cooperatives, as their initial experience suggested the previously established cooperatives were not functioning as planned due to frequent higher intensity conflict events between organizational members (e.g., anger, yelling, and physical altercations occurring at meetings, in social gatherings, and while in the field working).

This conflict between co-op members was explained to our research team by the NGO as being impacted by the extreme environment of historical conflict in the “Eastern corridor” of Northern Ghana, an area plagued with ethnic, tribal, and political turmoil despite the relatively consistent peace in the rest of the country. An additional aspect of Co-op Ghana's work was to create field research reports documenting the current state of conflict and development in the North. Therefore, we reviewed a series of secondary documents that provided quantitative and qualitative data confirming these claims and offering additional important contextual information. The eastern corridor has historically experienced intra- and inter-ethnic conflicts due to migration and settlement patterns. The conflict has intensified in the past three decades since the so-called guinea fowl war, sparked by a fight over the price of a guinea fowl in the market. This enduring conflict continues between ethnic groups primarily over chieftaincy battles (conflict over who is the rightful heir to a given chieftaincy domain), and land title disputes. Less frequent but equally disruptive are battles over political and religious beliefs, gender-based conflict, and conflicts over farmer versus pastoralist rights to land. Thus, this extreme environment of deep, unsolvable, zero-sum conflict between ethnic groups, tribes, genders, and religions aligns with the definition of an ICE.

These enduring conflicts have had devastating consequences on many aspects of life in this region. For example, frequent curfews initiated after violent incidents affect the literacy rate among children who are prohibited from going to school. Pregnant, elderly, and ill individuals have expressed reluctance to go to hospital for fear of being neglected or even deliberately given wrong medications by their rivals. Farming has been affected by vengeful acts of farm destruction and sniper attacks on farmers in their fields. People are afraid to increase livestock numbers for fear that recurrent violence will lead to their destruction. Trade is affected on both the supply-and-demand side: buyers do not go to certain markets for fear of being accused of patronizing rivals’ markets; meanwhile, sellers who have to travel at night or before sunrise to purchase wares are unable to do so for fear of being attacked in the dark or are forced to close their market stalls early because of curfews.

All members of the research team visited Northern Ghana to gain a deeper understanding of the ICE, as well as Co-op Ghana's existing efforts and the nature of the conflict events hindering them. During this trip we heard firsthand accounts of the ongoing unsolvable conflicts in the ICE and the specific conflict events involving cooperative members that had been documented in field reports. We were told about the historical tribal, ethnic, and political conflicts in their environment, as well as the extremely negative outcomes they experienced as a result of this intractable conflict. We also heard anecdotes of highly emotional conflict events (often involving weapons such as cutlasses) involving cooperative members over unpaid debts, access to the community well, and land boundaries. We even witnessed a highly emotional conflict event ourselves when a fight broke out among cooperative members during a meeting. Taken together, Co-op Ghana's secondary documents and our observations led us to conceptualize the environment as an ICE, and the conflict events the members of these cooperative organizations were experiencing as frequent and provoking very negative emotions, limiting their ability to successfully work together to accomplish the cooperative's goals. Moreover, this initial phase hinted to us that a prevention focus could be pervasive in this extreme environment, as the individuals frequently discussed the need to avoid negative outcomes. For a full timeline of the two years of this study, please see Appendix 1.

### Experimental Design, Procedure, and Sample

In designing and executing our study, we relied extensively on King, Hebl, Botsford Morgan, and Ahmad's (2012) prescriptions for undertaking field experiments on sensitive organizational topics. More specifically, we took great care with respect to (1) random assignment to conditions, (2) developing a research question that can be effectively measured, (3) getting consent and buy-in from key partners, (4) effectively training study participants, (5) ensuring construct validity of our treatment(s), (6) pilot testing of materials, (7) collecting reliable data, and (8) pairing the field experiment with other methods.

In the initial design process, we worked extensively with Co-op Ghana to develop an intervention that sought to improve the relations among cooperative members and thereby ultimately improve the success of newly formed cooperatives. More specifically, two versions of this intervention were designed, one focusing on “preventing/avoiding conflict” and the other focusing on “promoting/embracing peace” (see Appendix 2 for posters and Appendix 3 for exact wording). These different versions of the intervention were randomly assigned across 80 newly formed farming cooperatives: 40 cooperatives to the “prevention” condition and 40 cooperatives to the “promotion” condition. We elected not to assign any of the 80 newly formed cooperatives to a control group as (1) doing so would have further reduced statistical power in what was already a relatively small sample size, and (2) our research partner expressed concerns with the notion of withholding treatment that may offer benefit to the cooperatives seeking to help.

The 80 cooperatives represented all the newly formed cooperatives by Co-op Ghana within the year of this study. Furthermore, all 80 cooperatives were located in the Upper East region of Northern Ghana and overseen by Co-op Ghana's Bolgatanga satellite office within the region (their main office is 100 km south of the region in Tamale). Because the engagement between Co-op Ghana and the 80 cooperatives occurred in regular intervals, and within the community where the cooperative was located (rather than at the satellite office of Co-op Ghana), potential concerns regarding a confounding effect of physical distance were minimal.

As outlined in Appendix 1, the intervention design began with all coauthors traveling to Ghana to meet with the managers and field staff of Co-op Ghana, as well as to visit local individuals in communities of established cooperatives, as described previously. The purpose of these meetings was to better understand the specific nature of the ICE and the conflict events involving cooperative members, as well as to identify culturally specific behaviors that would be considered positive or negative. Upon returning from the field, this contextual information was subsequently combined with prior work on regulatory focus and regulatory fit theories to design the specific content and delivery for the intervention.

From a content perspective, each version of the intervention was designed to prevent/promote the respective behaviors based upon the specific condition. For instance, the “preventing negative activities” intervention outlined what behaviors should be avoided (e.g., “avoid distrust of others,” “avoid negative stories about others,” and “avoid violating others’ property”). In contrast, the “promoting positive activities” version of the intervention outlined the equal yet opposite behaviors that should be embraced (e.g., “embrace trust of everyone,” “embrace positive stories about everyone,” and “embrace respecting everyone's property”). With regards to the exact language used, the Gamache, McNamara, Mannor, and Johnson (2015) regulatory focus dictionary was used as a starting point, yet given our context, modifications were required in order to contextualize the language to the participants and their culture. To achieve this, we went through several rounds of revisions with Co-op Ghana's field staff on the types of words that captured these distinct ideas and together decided on the final wording used in the intervention. Additionally, given that there were multiple languages used within the region, great care was taken in the translation of all materials into the local dialects to ensure it was accurate and easily understandable. These translations were then translated back into English to ensure that the intended framing had been maintained in the local dialects.

The core intervention itself (which did not vary based on the version) initially consisted of a set of materials that walked participants through a “pledge,” whereby individuals pledged—at a pledge ceremony—to avoid conflict/promote peace in their cooperative and broader community. Co-op Ghana recommended that we augment this pledge with a song that members could sing to internalize the message. Our final set of materials consisted of (1) a colorful poster outlining the “pledge” accompanied with a group photograph of the cooperative members, (2) a document with the name and thumbprint of each cooperative member confirming their commitment to the pledge, and (3) a song containing the exact words of the pledge. The “prevention-framed” posters were colored red and green, and the “promotion-framed” posters blue and yellow to reflect culturally appropriate (as per Co-op Ghana's recommendations) “negative” and “positive” colors, respectively. To create the background music for the song, a musician/recording artist who was trained in West African drumming composed several alternative scores with rhythmic characteristics that are native to Sub-Saharan Africa to accompany the words in the pledge. The selection of the specific melody and beat was undertaken by Co-op Ghana field staff in accordance with what they felt would be best received by cooperative members. The melody and beat did not differ between the “prevention” and “promotion” conditions—only certain words changed. Two of the coauthors then returned to Ghana to work with Co-op Ghana's field staff to finalize the implementation of the intervention.

The pledge ceremony was conducted with each of the newly formed cooperatives by Co-op Ghana field staff. This involved (1) reading through the pledge, discussing its meaning and rationale behind it, and asking each cooperative member to “sign” it via thumbprint (for reasons of low literacy); (2) taking and attaching a group photograph to the large poster; (3) having the cooperative members sing the pledge song together; and (4) laminating and placing the poster in each cooperative meeting location, to be brought out at each of their weekly cooperative meetings, in order to reinforce the pledge. During the ceremony, members were encouraged to ask clarifying questions about the pledge, and it was suggested by the implementing field staff that it be sung at the start of subsequent cooperative meetings. An independent third-party was recruited by the coauthors to attend and observe the ceremonies to ensure their consistency. Subsequently, that same individual and one of the coauthors visited randomly selected cooperatives at intervals of approximately one month and four months following the ceremony to ensure that the posters were still in good condition and being prominently displayed and to confirm that the pledge was sung at the start of cooperative meetings.

### Data Collection and Measures

#### Dependent variable

Our outcome of interest was the number of conflict events that organizational members experienced over the nine-month period. The duration of nine months was chosen because (1) it allowed for measuring conflict events that occurred throughout an entire growing season where cooperatives met to coordinate with one another, and (2) it balanced the desire for examining longer-term effects and the risk of an unanticipated exogeneous shock within the Northern Ghanian region. The selection of an aggregate count of the “number of conflict events” over an extended period as our dependent variable made collecting a baseline measure for all 80 cooperatives logistically problematic. However, we did ask a single question to the community volunteers Co-op Ghana had assigned to each cooperative at their inception to give greater confidence that there were no significant a priori differences between the level of conflict in the 40 cooperatives assigned to the “prevention” condition and in the 40 assigned to the “promotion” condition. More specifically, just prior to the intervention, we asked each community volunteer to indicate the extent to which they agreed or disagreed (on a 7-point Likert scale) with the following statement: “Cooperative members have experienced a lot of conflict while working together.” Our analysis indicates that there did not appear to be any selection bias with respect to preexisting levels of conflict between the two conditions. Responses from the cooperatives assigned to the promotion condition had a mean level of conflict of 4.14, and those from the prevention condition had a mean level of conflict of 4.25—and most importantly, such differences were statistically nonsignificant (*p *= .760).

To subsequently track our dependent variable over the nine-month period, we created a conflict record sheet in collaboration with Co-op Ghana field staff that community volunteers were trained to complete following a conflict event (see Appendix 4). The community volunteer was trained to indicate the nature of the conflict event shortly after it occurred but were instructed not to record the names of cooperative members involved for the purpose of maintaining anonymity. Each conflict sheet was counted as a unique conflict event that occurred.

Each community volunteer had agreed to act as the local coordinator for all Co-op Ghana activities and were literate, well connected, and respected within their community. While there may indeed have been selection bias or demographic differences between community volunteers at the individual level, the use of random assignment would have distributed such heterogeneity relatively equally at the condition level and is therefore unlikely to systematically affect our statistical results. These individuals were not blind to their community's condition, but because of the remoteness of the area did not have communication across cooperatives.

To prepare volunteers to be able to recognize and record conflict events, a volunteer member from each community was provided with a two-hour training session by Co-op Ghana field staff approximately one month prior to the beginning of the intervention. As part of the training, community volunteers were provided with a series of role-playing exercises whereby the Co-op Ghana trainer would read aloud an example conflict event scenario, and the volunteer would be asked to subsequently fill out a conflict record sheet. After each scenario, a calibration discussion took place to address any questions or confusion. The training involved ensuring that volunteers would recognize and record every conflict event. There was not an inter-rater reliability assessment during the conflict tracker training session, as there was just one volunteer per community, and the training was done one-on-one between the Co-op Ghana trainer and the community volunteer. While again, this may have resulted in variance in the comprehensiveness of the training at the individual level, such variance is likely to have been evenly distributed at the intervention level through the process of random assignment and thus unlikely to have systematically affected our statistical results.

Although our focus was on the total number of conflict events, the conflict record sheet also asked volunteers to rate the “degree of verbal conflict” and “degree of physical conflict.” For verbal conflict, the scale ranged from 1, *Very Minor* (argue heatedly just short of yelling) to 5, *Very Major* (threats of death). For physical conflict, the scale ranged from 1, *Very Minor* (physical displays of aggression short of making contact, raised fists) to 5, *Very Major* (physical attacks causing severe physical injury: gunshot wound, severe cut, death). We measured severity of conflictual behavior to examine whether fewer conflict events masked greater severity.

Following their training, volunteers were provided with conflict record sheets to fill out when conflict events occurred over the next nine months. Co-op Ghana field staff collected the sheets from each of the community volunteers. At the conclusion of the nine-month period, 45 of the 80 volunteers (56% participation rate) provided information on conflict events involving cooperative members. Of this final sample of 45 cooperatives, 26 cooperatives were from the prevention condition and 19 were from the promotion condition. The results did not show a significant difference in attrition rates between our two conditions *B* = .719 (*SE* = .458), *p* = .117, highlighting that in both conditions volunteers were equally likely to participate in recording cooperative members conflict incidents. Additionally, the 45 communities that constituted our final sample did not differ from the 35 communities that did not track conflict incidents in terms of size (*p *= .907) or ethnic diversity (*p *= .541).

**Control Variables.** While randomly assigning cooperatives should collectively minimize the impact of alternative causal factors on the difference in the number of conflict events between the “prevention” and “promotion” conditions (Hill, Johnson, Greco, Boyle & Walters, 2021), there were two context-specific control variables that could potentially exert a disproportionate influence on these conflict events: (1) the number of cooperative members and (2) cooperative membership from more than one ethnic tribe. As a result, we included a control for size of cooperative (indicating the total number of members belonging to each cooperative), as well as a control for tribal diversity (whereby cooperatives with only a single tribe were coded 0 while those with more than one tribe were coded as 1). The data for these variables were obtained directly from Co-op Ghana's records that they maintained on the cooperatives.

## Findings

### Quantitative Results

A summary of the means, standard deviations, and correlations can be found in Table 2. No outliers were identified when examining reported counts of conflict events, and all observations from the 45 communities were kept in the data. All *p*-values reported are from two-tailed tests.

**Table 2:** Means, Standard Deviations, and Correlations^a^

	Mean	*SD*	2	3	4
Condition	0.578	0.500	−0.209	−0.147	0.165
Conflict Events	6.844	5.866		−0.249	0.132
Size of Cooperative	56.400	8.799			−0.152
Tribal Diversity	0.356	0.484			

^a^ *N* = 45. Condition (0 = Promotion-Frame; 1 = Prevention-Frame).

Using a generalized linear model within SPSS v27, we conducted a negative binomial regression to analyze the effect of our treatment conditions (0 = promotion; 1 = prevention) on the number of conflict events (with controls). A negative binomial approach is advocated over OLS regression when the dependent variable consists of count data (Blevins, Tsang & Spain, 2015). The likelihood-ratio test of alpha, which compares the log-likelihood values from a negative binomial and Poisson model (Long & Freese, 2006), was significant (χ^2^ = 64.828, *p* < .001), suggesting overdispersion on the outcome variable, making a negative binomial distribution more appropriate to use than a simpler Poisson distribution.

**Figure 2:**
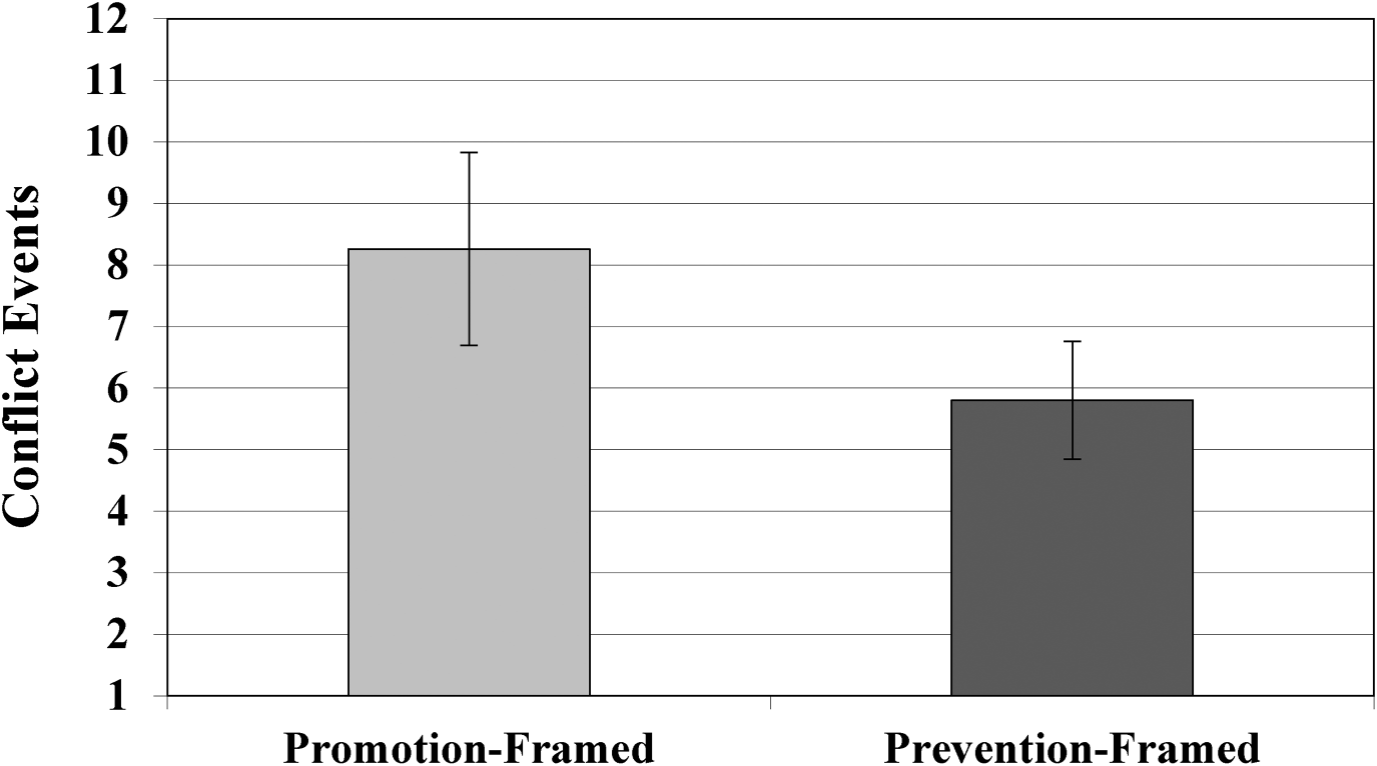
Number of Conflict Events Across Conditions (standard error bars shown)

When examining the number of conflict events, results showed that the two conditions differed significantly from one another: cooperatives assigned to the prevention condition had significantly fewer conflict events (*M* = 5.808) than cooperatives in the promotion condition (*M *= 8.263), (*B* = −0.516 [.230], *p* = .025) (see Figure 2). The Cohen's *d* effect size measure (i.e., standardized mean difference between conditions) was .423, indicating a moderate effect size for the prevention-framed intervention's association with fewer conflict events (see Table 3). That is, cooperatives in the prevention condition experienced 29.7% fewer conflict events than those in the promotion condition. The difference between these conditions supports the hypothesis that a prevention framing would be associated with fewer conflict events than a promotion framing.

**Table 3:** GLM Negative Binomial Regression (DV = Conflict Events)^a^

	*Β*	Std. Error	Wald Chi-Square
Condition	−0.516*	0.230	5.029
Size of cooperative	−0.028*	0.013	4.311
Tribal diversity	0.172	0.239	0.518

^a^ *N* = 45. Condition (0 = Promotion-Frame; 1 = Prevention-Frame). * *p *< .05.

Although the overall number of HCEs was less in the prevention condition than in the promotion condition, it is possible that these fewer instances may have masked more severe behaviors. That is, an alternative explanation for our findings is that cooperatives in the prevention condition were bottling up their conflicts and simply only expressing more severe behavior when they did experience an HCE. To examine this possibility, we first inspected cell means for ratings of severity of the behavior in the HCE. These means, pointed toward those in the prevention condition having less severe verbal (*M* = 2.007) and physical conflict (*M* = 1.529) than in the promotion condition for verbal (*M* = 2.303) and physical conflict (*M *= 1.857) as compared to the prevention condition. To test if these conditions differed in severity of conflict, we conducted supplemental linear regression analyses, where nonindependence of observations was accounted for by including a random intercept of cooperative ID (level 2) in a nested model, where the severity of conflict incidents (level 1) was the focal level of analysis. Although the prevention condition tended to have less severe instances of conflict, there were not significant differences across conditions in the reported severity of verbal, *B *= −.211 (.227), *p* = .357, or physical conflict *B* = −.163 (.185), *p* = .384. Taken together, these data do not support the alterative possibility that fewer HCEs in the prevention condition were indicative of more extreme or problematic outbursts.

### Additional Qualitative Triangulation

It has become commonly accepted best practice in field experiments to complement quantitative data with qualitative data that can confirm through triangulation or add nuance or depth to quantitative findings (Chatman & Flynn, 2005; King et al., 2012; Tashakkori & Teddlie, 2010; see, e.g., Grant, Berg & Cable, 2014; Kistruck et al., 2013; Slade Shantz et al., 2020; Vergne, 2012), particularly when researching sensitive (King et al., 2012) or contested (Roth & Mehta, 2002) organizational topics such as conflict events in an ICE. Therefore, our experimental design was “bookended” by significant time spent in the field by the authors collecting interviews; participating in focus groups; and observing before, during, and after the field experimental window. This iterative process of qualitative and quantitative analysis, with a field experiment as the central pillar, is referred to as “abductive experimentation” (Kistruck & Slade Shantz, 2022). An abductive approach to field experiments is well-suited to studying action-oriented interventions in partnership with organizations seeking to confront complex social issues (Aguinis, Ramani, & Villamor, 2019). Because complex social issues, often referred to as “Grand Challenges,” are typified by intertwined social, technical, and economic factors (Ferraro, Etzion, & Gehman, 2015), an abductive experimentation approach provides both the rigorous certainty of quantitative data and a more nuanced understanding of the environment that qualitative data can provide (Kistruck & Slade Shantz, 2022).

This approach begins with an initial inductive phase of knowledge assembly (Kistruck & Slade Shantz, 2022), which in this case involved a field visit by the entire research team, focused on gaining a deeper contextual understanding of the broader extreme environment and the conflict events that were occurring. This included meetings with Co-op Ghana and other peripheral organizations, as well as conducting focus groups in communities where Co-op Ghana was working. These were not formal interviews, but we took copious field notes of our observations, capturing some verbatim quotes by community members in them.

#### Existence of an ICE and Extreme Negative Outcomes

Our intent with this initial stage was to gain a more nuanced understanding of the nature of the enduring conflict environment in the region, increasing our certainty that this context did indeed fit the definition of an ICE. In this vein, many respondents relayed personal experiences of the ICE and its extremely negative outcomes, including physical violence. As such, we gained a better understanding, specifically, of the “intractability” of the ongoing tribal, ethnic, and political conflicts in their environment; the zero-sum natures of these conflicts; the individuals’ fear and anger toward the opposing parties; the extremely negative outcomes they have endured (including threats to their safety and identities); and the centrality of these conflicts in their lives. Specifically, cooperative members pointed to several aspects of the ICE in which they lived. Particularly, they noted the long-standing, deep, unsolvable tribal and ethnic conflict between the Nanumbas and Konkombas, whose members’ collective identity is based on their tribal membership. The resulting violence from this significant intractable conflict in their region caused them to flee their homes in fear for their own safety:

Binda is one of the oldest communities in Nanum and if you were going to count on the peaceful communities, Binda could not be counted [as] of them. The conflict between we Nanumbas and the Konkombas . . . when the whole thing happened, we run away to Bimbila and we stayed there for about two months or let me say three months before we came back.

#### Indication of a Prevention Focus Induced by the ICE

This next quote also demonstrates the enduring unsolvable conflict between tribes, which is seen as a zero-sum game, as one group's success threatens the survival of the others. In addition to discussing the aspects of the ICE and its extremely negative consequences in their lives, the cooperative members also indicated that this environment led to a strong focus on avoiding any negative events, even those unrelated to the enduring conflict—a prevention focus:

In the olden days there were some big conflicts between tribes, and it killed more of their tribes so this time they do not want any single thing to happen to create conflict. That is why they just don’t want conflicts in their communities.

#### Perception of Potential Conflict Incidents

Moreover, as theorized, interviewees talked about the *perception* of relatively minor potential conflict incidents, such as the use of a resource by another organizational member, as conflictual, leading to high-intensity negative emotions:

People were having an argument about yam seeds. The man said that these fellows went and took his yam seeds and there was about to cause some fight.

When the season of farming arrived, I was once sitting when my phone rang. When I picked the call one guy told me that someone had picked a tractor and was ploughing my farmland then I became annoyed. . . . When I went to the farm, I went with [a long knife], I am telling you.

These quotes illustrate that the perception of potential conflict incidents as conflictual occurred frequently in the ICE, resulting in high-intensity negative emotions. In contrast, if one farmer took another's seeds in a non-ICE, attribution of the negative event to the individual is less likely than in an ICE. Moreover, even if the farmer is perceived to have caused the unfair distribution of these scarce resources, the action is still more likely to be seen as unintentional in a non-ICE (i.e., the farmer was mistaken about whose seeds they were) than in an ICE.

#### Behavioral Differences from Interventions

Based partially on the insights gleaned from this field visit, the previously reported field experiment was designed, pilot tested, and executed. During the 9 months of data collection following the introduction of the intervention, one member of the author team conducted two qualitative data collection trips. The first was approximately two months after the pledge ceremony. During this trip, focus groups were conducted with eight communities (five prevention and three promotion) throughout the three districts visited. These focus groups were not recorded, but field notes were taken, with time directly following the focus groups dedicated to discussing observations with the research team.

Primarily, these focus groups were intended to identify any early qualitative examples of behavioral changes following the perception of potential conflict incidents as conflictual compared to the highly negative conflict events seen before the intervention started. These examples illustrate that following the introduction of the intervention, individuals’ behaviors changed in the prevention condition in a way that reduced the likelihood of a conflict event. In contrast, in the promotion condition, we see positive behaviors co-existing with conflictual behaviors but not crowding them out. Additionally, these quotes suggest a difference in the extremity of eager behavior (promotion condition) and vigilant behavior (prevention condition), which is again consistent with our theorizing of greater enthusiasm for preventing negative behavior under a prevention-framed than promotion-framed intervention. For example, we heard anecdotes from those in the prevention and promotion conditions, respectively, such as:

Initially when they were going to the farm, they always just go to the farm with cutlass, sometimes just to the farm but because of this intervention she went to the farm with her husband (without any weaponry).

I am a trader, and I sometimes enter into peoples’ homes when I go to sell things, and often men and women are fighting. I use the words of the pledge to help them resolve their differences.

#### Resonance of Preventing Negatives

In the final field visit, the same coauthor and a research assistant undertook a series of interviews. This field visit involved 106 interviews, across eleven communities, including both participants in the field experiment as well as nonparticipants, to gain a more holistic perspective. These interviews followed an informal interview guide, and our primary objective was to understand if, as we had theorized, the driver of our hypothesized results was indeed a higher level of resonance with the prevention framing and therefore a feeling of “rightness” for vigilant behavior to reduce the likelihood of conflictual behavior (conflict events). Here, our approach was to divulge our two conditions and quantitative findings in simplistic and general terms. Specifically, we explained that when we presented cooperatives with messaging around “duty,” “obligation,” and “preventing” something bad rather than “opportunity” and “promoting” something good, the “preventing” messaging seemed to resonate more. We then asked them why they thought that might be the case.

Our initial aim was to focus on the regulatory focus framing itself (prevention versus promotion) rather than the presentation of the goal as minimal versus maximal (avoiding conflict versus embracing peace). We did find suggestions that a prevention framing appeared to resonate more with the participants than the promotion framing, creating a “feeling of rightness” and as a result was more compelling:

Prevention is better than cure. You must prevent, than you say oh if I don’t prevent after I fall see I can just cure myself. No, you must prevent before you can cure, prevention is better than cure.

Prevention is the best. Because if you can prevent, there will be no need to sit and talk about peace.

People see it as a duty because always, anything you want to happen, we remember of that pledge that we have read, so when you think of it and remember of it you say that you will stop whatever you were going to go on about it.

Duty, because if they practice this it will prevent conflict. Because as they are all going and doing that, it will let them change themselves for them to stop conflict.

We did, however, also ask specific questions about presenting the goal as avoiding conflict versus embracing peace. Here, too, conflict seemed to resonate more than peace:

When you look at the 2 songs, the prevention of conflict, when they first sung it to them, it just entered straight into their heart.

Immediately you hear of conflicts, it tells you some bad things in your brain, are you getting it? It's very bad! When you hear of conflicts, no matter what it is, whatever conflicts, the name conflict it breaks the mind. So that is why preventing of conflict is more than the promotion of peace.

The latter quote seems to indicate that the environment, and the negative outcomes produced by it, are emotionally tagged in their brains, making them easier to recall and act upon, providing a vivid reflection of our theory as to why the prevention focus is induced by the ICE.

Two mechanisms (which we did not theorize but which appeared in our interviews) seemed to bolster this effect. We refer to these as *severity* and *sequence*. With regards to “*severity*,” we noted a difference in the strength or extremity of examples of conflict versus peace. For example, we did see evidence that the respondents understood the benefits of peace and the opportunities that came with it. At a basic level, peace seemed to bring opportunities for more social enjoyment. For example:

The way he knows there is peace in this community is he will come to my house and greet me. I will also go to his house and greet him. [In a conflict community] he will not come. I will not go to his house . . . so you will know there is no problem.

Respondents also spoke about opportunities for coordination and resource sharing that arise from peace. For example, such coordination can manifest as labor sharing among farmers:

That was peace in the community. We were organizing, we are all farming. Now let's say I want to farm 10 acres of land, and I have been low in my house, and I want to use May/June to do that work. How will I do it faster? And I don’t have money to invest. How will I do it? So we came together, and we agreed to start organizing communal labor.

Therefore, community members had some experience with the opportunities that arose from peace and understood that “embracing peace” was an attractive goal to strive to achieve. However, the prior experiences of interviewees with the threats arising from the ICE were comparatively much more severe, in terms of impact on their well-being, and therefore more salient and urgent to address, which further supports the theorized prevention focus in the ICE. Many respondents relayed personal experiences with physical violence that had previously taken place where communities were not able to “prevent conflict”:

As the borehole of the town . . . for getting water, for the women sometimes they quarrel, this one say, ‘I will kill,’ this one say, ‘I will kill’ and so they will fight. Every day they were fighting, every day like that.

At first, when conflict came between two people, they were coming out with the [long knife] and axes to beat one another. Someone and his brother were fighting. Very serious.

In the past when they fight with their wives you see they use the [long knife], others use gun . . . that I am going to kill you.

Additionally, we noted many respondents referring to a sequencing effect—that is, in such an extreme environment of intractable conflict the focus on avoiding negatives (prevention) is so strong that the reduction of conflict events is a required prerequisite before ideas about embracing peace can even be entertained. Thus, conflict events between the parties need to be addressed first to allow the individuals to be able to focus on promoting positives (promotion). This observation also lends support for the inability of the promotion-framed intervention to situationally induce a promotion focus in the ICE. For example, respondents stated:

It's like climbing a ladder, you have the first step, the second one, before, it's a process. So I think preventing, is the first thing we look at. If you are able to prevent it you can promote it.

So if you prevent conflict, it's far better than you let conflict before you come and talk of peace. Don’t let the conflict to come before you come and talk about conflict. Don’t let conflict to come, before you must come and talk about make peace, no you don’t do that so ours is talking about prevent conflict. When you prevent conflict, then there will be peace. But without preventing conflict, peace will not be there. If I fall or if I didn’t wash my hand and if I fall then they will cure me, NO, preventing is better than cure.

Before you ensure peace, we have to prevent it (conflict). And we also have a saying, you can’t give people what you don’t have. You only give people what you have. So if we are able to prevent conflict . . . then we can actually talk to people about peace. So we need to prevent it, before we can promote it. That is why I actually think people embrace the prevention more than the promotion.

Together, our qualitative evidence suggests that not only were our participants in an environment of intractable conflict, but that this environment seemed to induce a prevention focus. Additionally, while organizational members understood the benefits of peace and saw it as desirable, their extremely negative experiences with intractable conflict emotionally tagged these outcomes, contributing to the induction of the prevention focus by this environment. Finally, we saw evidence that the prevention focus increased the perception of potential conflict incidents as conflictual and created a greater resonance with prevention of conflict events and vigilant behavior as a means of achieving it. Thus, the qualitative findings supported our theorizing underlying the impact of an intractable conflict context on how even a relatively minor potential conflict incident may be perceived by organizational members as conflictual and the higher level of resonance between their lived experience in the ICE and a prevention-framed intervention.

## Discussion

In this paper, we explore the impact of framing on ex ante interventions to address conflict events involving organizational members in an ICE. We find that a prevention-framed intervention is associated with fewer conflict events than a promotion-framed intervention in this extreme environment. We argue this occurs in our study because the prevention focus induced by the ICE matches the prevention-framed intervention, increasing the likelihood that individuals in these cooperatives reduced conflict behaviors. This finding contrasts with prior work outside of ICEs, which suggested promotion-framed interventions are more successful at reducing conflict by shaping the perception of potential conflict incidents (Weber & Mayer, 2011). Specifically, outside an ICE, a promotion-framed intervention situationally induces a promotion focus, leading to a view of the other party as cooperative and the experience of more positive emotions, all of which color the perception of the event in a positive light (e.g., Weber & Bauman, 2019).

In contrast, we argue that an ICE induces a prevention focus, arising from the activation of strongly encoded memories of extremely negative outcomes by salient environmental threats. Moreover, given that the salience of this environment and its extreme threats are much higher than that of the promotion framing from the intervention, a situational induction of a promotion focus is unlikely to occur. As a result, we suggest this novel effect arose from the “fit” between the prevention framing of the intervention and the organizational actors’ strong prevention focus, which makes vigilant behavior feel “right,” reducing conflict events. We saw indications of this theorizing in our interviews, as individuals suggested that an ICE invokes a strong focus on avoiding negative outcomes and a high resonance of the prevention-framed intervention.

### Conflict Events in an Intractable Conflict Environment

Our study makes several contributions. First, we identify that the intervention that most effectively addresses conflict events ex ante in an ICE is the opposite of that prescribed in non-ICEs, such as those typically studied in organizational behavior and strategy. Relatedly, we expand understanding of the impact of an extreme external environment of intractable conflict—an ICE—on organizational conflict events. Specifically, we show how an ICE outside of the organization can shape members’ perception of a potential conflict incident inside of the organization. In doing so, we suggest the prevention focus induced by an ICE increases the likelihood that a potential conflict incident is seen as conflictual, and that this perception induces more high-intensity negative emotions than in a non-ICE. Typical organizational conflict events were conceptualized in a non-ICE as a temporary “outcropping in an otherwise smooth flow of a stable and cooperative set of relationships that made up the organization” (Pondy, 1992: 257). While this view was updated over time with a more dynamic perspective, where longitudinal studies show patterns of conflict events as they change over time (e.g., Jehn & Mannix, 2001), the fundamental assumptions about the emotionality of organizational conflict events has largely remained constant in this literature (Volkema & Bergmann, 1989). That is, “power, violence, dissolution or revolution might occur between nations, or gangs, or social classes, or within troubled families, but not within those islands of sanity and purposiveness called formal organizations” (Pondy, 1992: 257). By expanding the contexts in which organizational conflict can occur, we can expand the emotional spectrum of organizational conflict events to include even highly emotional conflict events in an ICE. As a result, the literature can better address organizational conflict across these different environments.

Moreover, this study expands our understanding of the impact of framing in the cognitive governance literature by employing a regulatory fit theory lens. While prior work has focused on framing to situationally induce a particular regulatory focus, we argue that an ICE will prevent this induction from occurring. In this case, we show that a frame matching that of the regulatory focus induced by the extreme environment is more effective at addressing conflict events.

### An Environmentally Induced Regulatory Focus

Second, we contribute to regulatory focus and fit theories by incorporating the broader environment within which dispositional (chronic tendency) and situational (induced by momentary cues) regulatory focus occurs. As such, our study suggests the possibility of a novel concept, an *environmental regulatory focus* that is theoretically distinct from dispositional and situational regulatory focus (see Table 4 for a summary of these differences). Dispositional regulatory focus differs across people, with each person having a chronic prevention focus, promotion focus, or a tendency to switch between them (Higgins, 1998). Thus, not all individuals in the same environment or situation will have the same dispositional orientation (Wallace, Little, Hill, & Ridge, 2010), as it depends on interactions with their caregivers in childhood. In contrast, the cues from an extreme environment or a momentary situation may be salient enough to induce a regulatory focus that temporarily overrides their dispositional regulatory foci, leading to a provisional common regulatory focus across them. That is, the ability of situational or environmental regulatory focus to override dispositional regulatory focus arises from the greater salience of momentary situational or environmental cues than that of a general dispositional tendency (Pham & Higgins, 2005).

**Table 4:** Contrasting Dispositional, Situational, and Environmental Regulatory Focus

	Dispositional	Environmental	Situational
Source	Childhood interactions with caregivers	Characteristics of an extreme environment	Framing from momentary situation
Duration	Chronic	When in environment	Short
Strength	Weak	Strong	Medium
Hierarchy	Dominated by situational and contextual	Dominates dispositional and situational	Dominates dispositional but dominated by contextual

However, situational regulatory focus and environmental regulatory focus arising from an extreme context also differ. Cues from an extreme negative environment are likely to be much more salient than those from a momentary situation, as memories of negative outcomes in the extreme environment are emotionally tagged. Thus, a situational induction of regulatory focus is unlikely to overcome an environmental regulatory focus from an ICE even temporarily, as the extreme negative environmental cues are more likely to fully engage the individual's attention. Additionally, an environmental regulatory focus from an extreme negative environment is more likely to be sustained while the individual remains in it, unlike a short-lived situational focus. Thus, we suggest that the induction of regulatory focus from an extreme negative environment lasts longer than a momentary situational induction. Moreover, dispositional regulatory focus and environmental regulatory focus may or may not be correlated, because not all life events shape dispositional regulatory focus, only experiences in the caregiver-child relationship (Higgins, 1998). For example, a child raised by caregivers who focus on rewarding positive behavior may have a promotion dispositional regulatory focus. However, they may later be thrust into the environment of a prolonged war, creating a prevention environmental regulatory focus.

In summary, we suggest that an environmental regulatory focus arising from an extreme environment may be unique in that it is (1) derived from a different source than either dispositional or situational regulatory focus, (2) stronger and more resistant to being overridden by another type of regulatory focus, and (3) longer lasting while the individual remains in the environment. The identification of this concept may change prior literature in significant ways. An environmental regulatory focus would fundamentally change RFT because it allows for a shift of an individual's goal orientation away from their dispositional focus for a more prolonged period than a momentary situational induction. Additionally, this concept may help to bridge the disparate framing literatures in organizational theory and psychology. On the one hand, the organizational theory literature examines framing from a macro perspective (Cornelissen, Durand, Fiss, Lammers, & Vaara, 2015; Kennedy & Fiss, 2009; Rhee & Fiss, 2014). On the other hand, psychology takes an individual perspective, whereby frames are used as primes (Lount et al., 2017; Molden, 2014). We combine these perspectives to suggest that environments can trigger a particular regulatory focus, which in turn can affect organizational outcomes.

Similarly, prior research suggested work environment can induce a particular RFT (Johnson, Smith, Wallace, Hill, & Baron, 2015; Wallace, Johnson, & Frazier, 2009). However, while the workplace can be a strong environment, it is not an extreme environment and can be overridden by situational-induced regulatory focus (e.g., Weber & Bauman, 2019). Thus, prior examinations of environmental influences on regulatory foci have also faced a range restriction that prevents a more nuanced understanding of how to craft interventions to shape organizational members’ perceptions in a particular way or to increase feelings of “rightness” for desired behaviors. This study allows us to go beyond these range restrictions to address the impact of much more extreme environments and their implications for conflict event intervention design.

While our study allows for the possibility of an environmental regulatory focus, we only examined an ICE, which is an extreme negative environment with highly salient negative cues that are likely to be emotionally tagged in the brain and yield a strong prevention focus. Yet, the possibility exists that an extremely positive environment may induce a strong promotion focus. While we believe that our predictions in Table 4 will hold, it is necessary to empirically examine this case to determine the relative strength of induction of a situational versus an environmental regulatory focus under these different conditions.

### Contributions to Practice

Finally, we provide theory-based guidance for managers to create interventions to address conflict events in the extreme environment of intractable conflict. This is particularly important because traditional interventions to address organizational conflict are less likely to be successful. Additionally, conflict events in ICEs are extremely harmful to organizations due to their high negative emotionality. Thus, this implication is particularly important, as it could serve to make workplaces safer for employees and, at the extreme, even save lives.

In this vein, this paper also specifically contributes to the growing literature on the importance of cooperatives for local economic development in areas characterized by intractable conflict, such as rural Africa (e.g., Khumalo, 2014; Okem & Stanton, 2016). Specifically, it highlights the impact that an ICE has on the frequency and intensity of organizational conflict events in these organizations. It also offers new tools for potentially reducing these more intense emotional conflict events involving organizational members, which not only differ from, but are more effective than, those traditionally prescribed in Western management theory.

However, our findings are not only important for efforts to alleviate poverty in the Global South. They are also important for reducing conflict events in organizations in wealthy nations, as ICEs are becoming much more prevalent in the Global North, and as a result even typical organizational conflict events have become increasingly emotional due to polarization and extremism. For example, in the United States the increasing perception of conflict in organizations (Ontic State of Protective Intelligence Report, 2022) may stem from an unprecedented level of partisan division (Newport, 2019), which led to an armed insurrection at the U.S. Capitol as lawmakers gathered to certify the 2020 Electoral College votes. Additionally, the United States is experiencing increasing participation in movements and protests around emotionally laden topics (e.g., pro-life versus pro-choice abortion rights, Black Lives Matter supporters versus Blue Lives Matter, maskers versus anti-maskers, vaxxers versus anti-vaxxers, climate advocates versus climate change deniers) and increasing economic disparities. While these divides have previously existed, they have been laid bare by the confluence of increasing polarization in all aspects of life, the popular movement toward authenticity in work (Fiol et al., 2009), along with the rising popularity of work cultures that encourage bringing ones’ “whole self” to work. This divide has been further intensified by the echo chamber effect on social media platforms (in which one's views are confirmed while opposing views are hidden or deleted). Whereas these differences were previously kept out of the workplace, people today find themselves working side-by-side in their organization with others who have visible opposing ideological views, against a national and international backdrop of increasing hostility and negativity toward others with differing views (Newport, 2019).

### Limitations & Future Research

In addition to those that we have already discussed (inability to measure pretreatment conflict levels, lack of a control group, and the possibility of coder bias), we recognize other limitations in our study. First, we were not able to directly measure the organizational members’ regulatory foci in the field. While situational regulatory focus is generally primed in a laboratory, it is difficult to test whether an ICE induces a prevention focus in farming cooperatives in Ghana due to literacy issues. Additionally, to examine the distinctiveness of environmental RFT, it would be interesting to examine an individual's regulatory focus both inside and outside of an ICE, which was not possible in Ghana, as the field experiment was conducted in an enduring ICE and participants did not travel far from their villages into the non-ICE portion of Ghana. Instead, we augment our field experiment with a qualitative examination of regulatory focus in an ICE.

It would also have been powerful to observe whether an environmentally induced or situationally induced regulatory focus (via the framed intervention) dominated. Although the greater success of the prevention-framed intervention as compared to the promotion-framed intervention suggests that the environmentally induced prevention focus dominated, future studies should be done in strong natural environments without these constraints, such as inside an established organization in a developed environment with divisions in both an ICE and a non-ICE. Additionally, laboratory studies could be constructed to compare the relative strength of an individual's chronic regulatory foci, situationally induced foci, and environmentally induced foci between ICE and non-ICE environments.

Moreover, to disentangle the effects of an ICE from an impoverished context such as the eastern corridor of Ghana, as well as its uniqueness in terms of cultural and institutional norms, future work might attempt to replicate this study's findings in an ICE in a different context. For example, areas outside of abortion clinics, political debates, and protests may represent intractable conflict environments in developed countries. Additional laboratory or field studies could also be undertaken to examine heterogeneity *across* ICE environments as it relates to prevention vs. promotion framing on conflict to gain greater insight into the nuances and points of inflection beyond the dyadic approach of this study.

Furthermore, we were not able to explicitly measure individual responses to the interventions but instead had to rely on an aggregate count of conflict events involving cooperative members. Again, in another environment, without the issues that we faced in our field study, the individual impact of differently framed interventions on conflict events involving organizational members in an ICE could be observed.

Future work may also examine the relative benefits of prevention and promotion-framed interventions relative to no intervention (i.e., a pure control). Although we posited prevention framing would be associated with fewer conflict events than promotion framing and found a moderate effect size, there may be an even larger positive effect size compared to a pure control. Moreover, given prior work in non-ICEs have documented that promotion-framed interventions are helpful, it may be the case that relative to a pure control, it may have still been associated with fewer conflict events. Additionally, we did not focus on how a “prevention” framing versus “promotion” framing may lead to differences in the frequency of conflict incidents over time. It is possible that different interventions may result in different temporal patterns in conflict (logarithmic, exponential, u-shaped) that may provide further insight into their relative efficacy.

## Conclusion

In conclusion, this study addresses a long-standing concern in organizations in emerging economies, as well as a growing concern among organizations in wealthy nations—addressing organizational conflict events in ICEs. We shed light on why traditional organizational conflict and cognitive governance interventions are less likely to address conflict events in an ICE. Instead, we illustrate that contrary to a non-ICE, a prevention-framed intervention is likely to be most efficacious at addressing conflict events in this extreme context. Thus, rather than “giving peace a chance” in organizations in an ICE, we suggest that the C-suite tackle this important strategic issue by “giving conflict avoidance a chance.” As illustrated in our study, this unconventional approach may make the workplace safer and even save lives in an ICE.

## APPENDIX 2

“Prevention”^a^ and “Promotion”^b^ Posters^c^

^a^Dark gray = green, light gray boxes = off-white with red outline. Text = black

^b^Dark gray = yellow, white boxes  = white with blue outline. Text = black

^c^“Our Cooperative” box = photo of the group, “My Obligation”/“My Opportunity” boxes = names and thumbprints of members

## APPENDIX 3

**“**Prevention” and “Promotion” Pledges[Table table6-01492063231196556]

**Table table6-01492063231196556:**

**Prevention Pledge**	**Promotion Pledge**
**Preventing Conflict** It is my obligation to PREVENT conflict in my community and cooperative. Stop negative THOUGHTS preventing unity AVOID distrust of others AVOID focusing on differences AVOID negative thoughts of others It is my obligation to PREVENT conflict in my community and cooperative. Stop negative WORDS preventing harmony AVOID intimidating others AVOID negative stories about others AVOID heated words It is my obligation to PREVENT conflict in my community and cooperative. Stop negative ACTIONS preventing peace AVOID violating other's property AVOID using weapons AVOID violent actions	**Promoting Peace** It is my opportunity to PROMOTE peace in my community and cooperative. Increase positive THOUGHTS promoting unity EMBRACE trust of everyone EMBRACE focusing on commonalities EMBRACE positive thoughts of everyone It is my opportunity to PROMOTE peace in my community and cooperative. Increase positive WORDS promoting harmony EMBRACE encouraging everyone EMBRACE positive stories about everyone EMBRACE peaceful words It is my opportunity to PROMOTE peace in my community and cooperative. Increase positive ACTIONS promoting peace EMBRACE respecting everyone's property EMBRACE everyone's markets EMBRACE peaceful actions

## APPENDIX 4

Conflict Record Sheet[Table table7-01492063231196556]

**Table table7-01492063231196556:**

**Degree of Verbal Conflict** (please check one): ___1 Very Minor (Argue heatedly but short of yelling) ___2 Minor (Angry yelling) ___3 Moderate (Swearing, personal insults, and/or abusive words) ___4 Major (Threats of retaliation physical violence) ___5 Very Major (Threats of death)	**Degree of Physical Conflict** (please check one): ___1 Very Minor (Physical aggression without contact—aggressive pointing, raised fists ___2 Minor (Physical attack without causing injury—striking, kicking, pushing) ___3 Moderate (Physical attack causing moderate injury—bruises, sprains, welts) ___4 Major (Physical attack causing major physical injury—broken bones, deep lacerations) ___5 Very Major (Physical attacks causing severe physical injury—gunshot wound, severe cut, death)
